# Missed Diagnosis of a Rare Retropatellar Juxta-Articular Angioleiomyoma: A Case Report

**DOI:** 10.7759/cureus.49182

**Published:** 2023-11-21

**Authors:** Kassem Ghayyad, Ali Īhsan Kiliç

**Affiliations:** 1 Orthopedic Surgery, Rothman Orthopaedics Florida at AdventHealth, Orlando, USA; 2 Orthopedic Surgery, İzmir Bakırçay University, Izmir, TUR

**Keywords:** retropatellar, surgical case reports, arthroscopy, angioleiomyoma, juxta-articular

## Abstract

Angioleiomyomas are benign tumors that originate from smooth muscle cells and most commonly affect organs such as the uterus or gastrointestinal tract. This article presents a case of a rarely reported angioleiomyoma located in the retropatellar juxta-articular region of the knee. The patient is a 42-year-old female who experienced chronic anterior knee pain that led to two unsuccessful arthroscopic surgeries. Magnetic resonance imaging (MRI) revealed a well-defined lesion in the retropatellar area, prompting the decision to proceed with open surgery. The histopathological examination confirmed the diagnosis of angioleiomyoma. This case highlights the challenges in diagnosing angioleiomyomas in the knee and emphasizes the importance of comprehensive MRI evaluation for accurate diagnosis and appropriate surgical intervention. Prompt identification and excision of the soft tissue lesion can lead to the complete resolution of symptoms and effective management of this rare condition.

## Introduction

An angioleiomyoma is a benign tumor that develops in the soft tissues and originates from smooth muscle cells [1]. Angioleiomyomas are relatively uncommon among benign tumors in soft tissues, accounting for only 4.4% [2]. Moreover, when excluding those that originate in cutaneous and subcutaneous tissues, angioleiomyomas in the extremities are exceptionally rare [3].

The preoperative diagnosis of angioleiomyoma is often uncommon due to its rarity and limited awareness among orthopedic surgeons in this field. As a result, patients may undergo multiple rounds of conservative or surgical treatments, particularly when the neoplasm is located near the knee joint [4-6]. In cases where these rare tumors are situated in the knee, misdiagnosis with more common pathologies such as meniscus or cartilage diseases can occur as observed in the patient described in this case report.

This case report focuses on a patient with angioleiomyoma located in the retropatellar juxta-articular region of the knee. To the best of our knowledge, no similar cases have been reported in the existing literature.

## Case presentation

A 42-year-old female patient presented to our clinic with a history of chronic right anterior knee pain persisting for approximately five years. Initially, the pain was sporadic and nonspecific. However, the patient reported having trouble while climbing stairs and kneeling due to pain. She localized the pain just above the tibial tubercle and described it as being aggravated by light touch. The patient denied any history of trauma. She mentioned that she had undergone arthroscopic knee surgery twice in 2019 and 2021 for the same chief complaint, without resolution of symptoms. Unfortunately, surgical reports from her previous surgeries were unavailable.

On physical examination, full extension of the knee joint was observed, but active flexion was limited (-30 degrees) due to pain. No noticeable swelling or redness was observed around the knee joint. Light touch palpation elicited tenderness in the area just above the tibial tuberosity. Furthermore, there were no signs of muscle atrophy, typical indications of meniscus lesions, or any evidence of ligament injuries.

**Figure 1:**
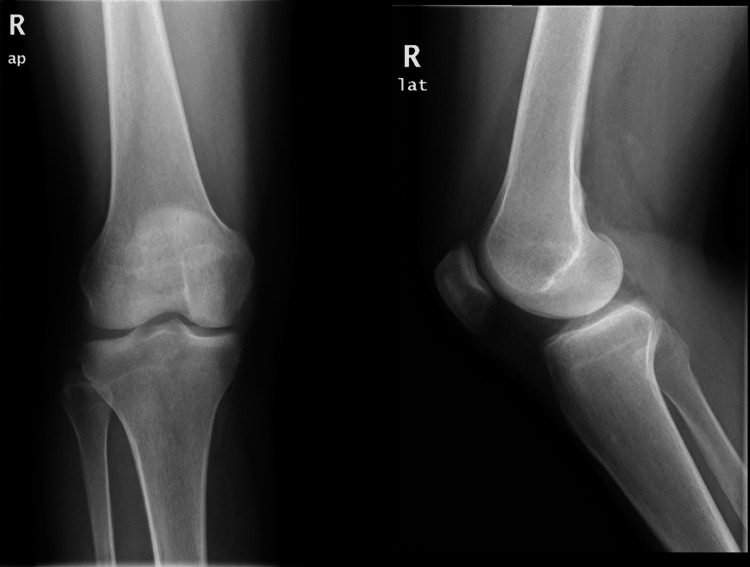
Anteroposterior and lateral X-ray views of the knee. No abnormalities were detected in the bone and soft tissues.

The knee MRI image obtained before the patient's initial knee arthroscopy surgery and the current knee MRI image were meticulously examined and compared. In both MRI scans, a distinct and well-circumscribed lesion was identified in the retropatellar juxta-articular region. Plain orthogonal radiographs of the knee did not reveal any abnormalities in the bone or soft tissue (Figure 1).

**Figure 2:**
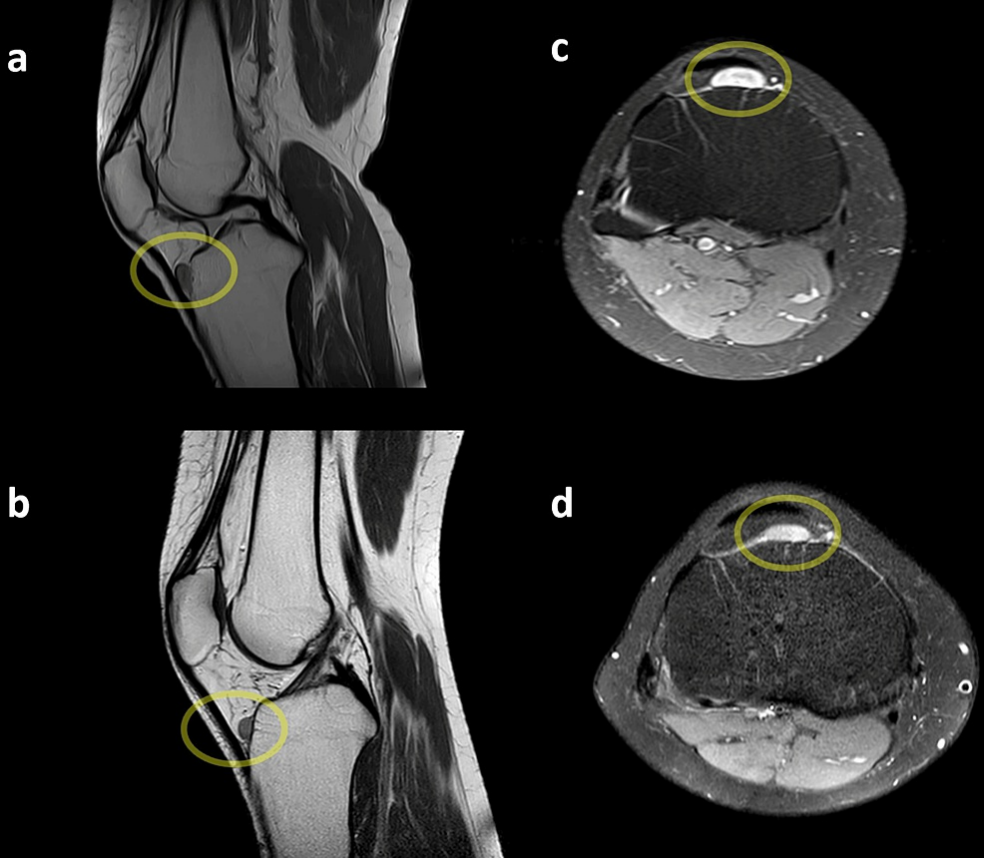
(a, c) MRI scan obtained before the patient's first surgery in 2019; (b, d) patient's current MRI scan in 2021. (a, b) In the sagittal view, T1-weighted MRI scan, the leiomyoma appears as a well-demarcated soft tissue mass with intermediate signal intensity, displaying a homogeneous nature. (c, d) In the axial view, T2-weighted MRI scan, the lesion is observed as hyperintense, indicating a brighter signal, and exhibits a well-circumscribed appearance.

The lesion appeared hyperintense on T2-weighted images and hypointense on T1-weighted images (Figure 2).

**Figure 3:**
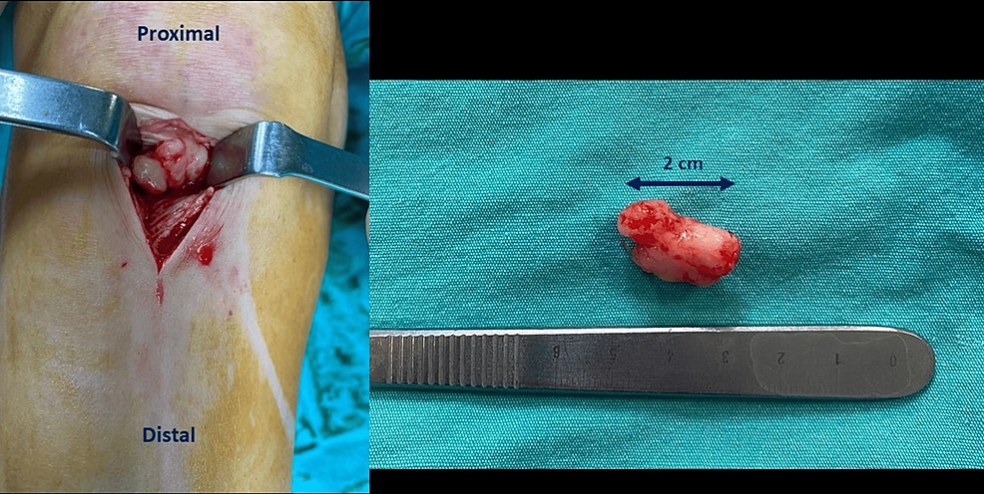
Intraoperative views revealed a solid mass measuring 2 cm x 1 cm x 0.5 cm

Based on these diagnostic findings, the decision was made to proceed with surgery. Due to the previous unsuccessful arthroscopic surgeries and the challenges associated with completely excising the lesion using an arthroscopic approach, the patient underwent open surgery (Figure 3).

**Figure 4:**
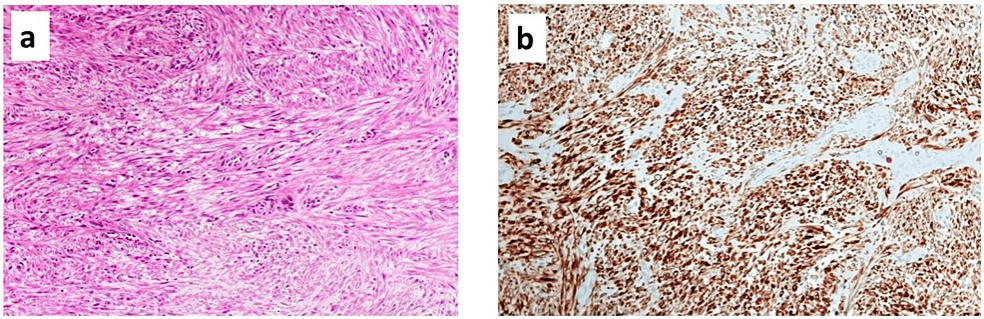
(a) The leiomyoma specimen shows a section composed of intersecting fascicles of spindle cells with uniform cigar-shaped nuclei (H&E, 200x magnification). (b) Immunohistochemical staining demonstrates desmin positivity in the leiomyoma (200x magnification). H&E: Hematoxylin & eosin.

As revealed in the pathology report, the specimen appeared as a medium-hard, nodular material with a smooth surface macroscopically. It measured 2 cm x 1 cm x 0.5 cm and had a solid, off-white whorled appearance on the cross-sectional cut. Microscopically, the lesion was well-defined and characterized by intersecting fascicles of spindle cells with uniform cigar-shaped nuclei. Immunohistochemical analysis reveals positive staining of these cells for smooth muscle markers including desmin, smooth muscle actin, caldesmon, and calponin. Microscopic findings reveal that the tumor comprises numerous blood vessels (hematoxylin and eosin, x20, x40). The final diagnosis was a benign angioleiomyoma with clean margins free of cellular atypia (Figure 4).

## Discussion

Angioleiomyoma is a benign tumor that typically arises from smooth muscle cells and primarily affects organs containing smooth muscles, such as the uterus or gastrointestinal tract. When it occurs in soft tissues, it usually involves the dermis and subcutaneous layers, with deep soft tissue involvement being extremely rare [2]. Depending on its location, it can be classified as cutaneous leiomyoma, angioleiomyoma, or leiomyoma of the deep soft tissue [1]. The first comprehensive review of this rare lesion was published by Stout [7] in 1937, and it has been well-documented in the literature ever since. While these tumors are observed in the lower extremities, only a few reports are available in the orthopedic literature [4,5].

Hachisuga et al. [2] conducted a study involving 562 cases of leiomyomas, revealing that these tumors primarily occur in individuals between the fourth and sixth decades of life. Approximately 90% of cases affect the extremities, with the lower extremities accounting for around 80% and displaying a higher prevalence among females. In contrast, head and upper extremity lesions are more common in males. Pain and tenderness are the most frequently reported symptoms, which are observed in 60%-75% of patients. Solid histologic subtype and lower extremity lesions typically present as painful subcutaneous masses, often exhibiting well-localized pain, exquisite tenderness, and temperature sensitivity. These symptoms overlap with glomus tumors, and histologic evaluation is necessary for differentiation. Besides glomus tumors, the differential diagnosis for small painful extremity lesions includes hemangiomas, angiolipomas, ganglions, neurilemomas, traumatic neuromas, and eccrine spiradenoma [1].

Magnetic resonance imaging (MRI) plays a valuable role in the diagnostic process as it is a noninvasive imaging technique that helps localize the tumor and rule out other potential causes of knee pain. The presence of post-gadolinium enhancement on MRI indicates the vascular nature of the lesion [8]. However, the definitive diagnosis can only be confirmed through histopathological examination of the tissue specimen.

Anterior knee pain is a common complaint characterized by discomfort in the front of the knee, typically around or behind the patella. It is frequently associated with conditions like patellar chondromalacia, patellar tendinitis, and bursitis [6]. While these are commonly encountered causes, it is important to consider rare cases as well. In the presented case, the patient has been experiencing anterior knee pain for approximately five years and has undergone two previous arthroscopic surgeries for the knee for this reason. However, both the old and current MRI reports lack a diagnosis or description of the lesion. The location of the lesion in the retropatellar juxta-articular region may pose challenges for arthroscopic excision. Despite the operations the patient underwent, it was observed that the retropatellar lesion was present in both old and current MRI images. These findings led us to think that the patient had undergone redundant arthroscopic surgery. Unfortunately, the surgical and pathology reports from the previous arthroscopic surgeries were unavailable. Due to the patient's history of undergoing two previous arthroscopic surgeries and the challenges associated with arthroscopic excision of the lesion, the decision was made to perform open surgery.

## Conclusions

A careful MRI examination for a suspicious mass lesion is crucial for achieving an early and accurate diagnosis of angioleiomyoma, the cause of anterior knee pain in this case. Excision of the well-circumscribed soft tissue lesion not only provides a histopathological diagnosis but also results in the complete resolution of symptoms as demonstrated in the present case. This emphasizes the importance of prompt identification and surgical intervention in managing this condition effectively.
